# Functional Roles of Gastrin-Releasing Peptide-Producing Neurons in the Suprachiasmatic Nucleus: Insights into Photic Entrainment and Circadian Regulation

**DOI:** 10.1523/JNEUROSCI.0065-25.2025

**Published:** 2025-05-22

**Authors:** Ruoshi Li, Ran Inoue, Hisashi Mori, Arisa Hirano, Takeshi Sakurai

**Affiliations:** ^1^Institute of Medicine, University of Tsukuba, Tsukuba, Ibaraki 305-8575, Japan; ^2^International Institute for Integrative Sleep Medicine (WPI-IIIS), University of Tsukuba, Tsukuba, Ibaraki 305-8575, Japan; ^3^Department of Molecular Neuroscience, Faculty of Medicine, University of Toyama, Toyama 930-8555, Japan; ^4^Life Science Center for Tsukuba Advanced Research Alliance, University of Tsukuba, Tsukuba, Ibaraki 305-8577, Japan

**Keywords:** circadian rhythms, gastrin-releasing peptide, gastrin-releasing peptide-producing neurons, photic entrainment, suprachiasmatic nucleus

## Abstract

The suprachiasmatic nucleus (SCN) serves as the central circadian clock in mammals, coordinating daily rhythms in both behavior and physiology. In the SCN, gastrin-releasing peptide (GRP)-producing neurons (GRPNs) are predominantly located in the core region, suggesting their possible involvement in photic entrainment. However, the specific contribution of GRPNs to the regulation of circadian rhythms remains poorly understood. This study utilized a Cre-driver mouse line, *Grp-iCre* knock-in (KI) mice, in which Cre recombinase is exclusively expressed in GRPNs, allowing the selective manipulation of SCN GRPNs to investigate their characteristics and functional roles in circadian regulation. All experiments were conducted in adult male mice. Anatomical tracing revealed that SCN GRPNs primarily project to the thalamus and hypothalamus, whereas input mapping demonstrated that SCN GRPNs receive most synaptic inputs from within the SCN. Behavioral analyses revealed that neither GRP deficiency nor ablation of SCN GRPNs significantly affected circadian locomotor activity rhythms or photic entrainment. However, chemogenetic stimulation of the SCN GRPNs is sufficient to induce phase shifts in behavioral rhythms. Additionally, calcium imaging with fiber photometry indicated that SCN GRPNs quickly responded to photic stimulation, with increased neural activity following retinal exposure to white light. These findings suggest that SCN GRPNs play a role in photic entrainment, albeit potentially redundant with other neuronal populations such as vasoactive intestinal peptide-producing neurons.

## Significance Statement

The suprachiasmatic nucleus (SCN) functions as the central circadian clock in mammals, synchronizing internal rhythms with the external light/dark cycle. Among its diverse cell types, gastrin-releasing peptide (GRP)-producing neurons (GRPNs) have been implicated in light-based entrainment, but their specific roles remained unclear. Using targeted genetic tools, we demonstrated that these neurons respond rapidly to retinal light stimulation and, when artificially activated, can induce phase shifts in behavioral rhythms. However, eliminating these neurons does not disrupt circadian behavior or molecular clock rhythms, indicating functional redundancy within the SCN network. Our findings clarify the modulatory—but nonessential—role of GRPNs in light-induced entrainment and underscore the complexity and resilience of circadian circuit organization.

## Introduction

The suprachiasmatic nucleus (SCN) serves as the central circadian pacemaker in mammals and coordinates daily behavioral and physiological rhythms. Within the SCN, diverse neuronal populations engage in complex intercellular communications to sustain these rhythms (Reppert and Weaver, 2002). Gastrin-releasing peptide (GRP)-producing neurons (GRPNs) are predominantly localized to the SCN core, a key site for receiving photic input from the retina (Antle et al., 2005). GRP and its receptor, the gastrin-releasing peptide receptor (GRPR), are considered pivotal in the phase resetting of circadian rhythms. In vitro studies have shown that GRP induces phase shifts in SCN firing (McArthur et al., 2000) and PER2 expression rhythms (Gamble et al., 2007). In vivo studies have also demonstrated that GRP administration upregulates c-Fos (Antle et al., 2005; Piggins et al., 2005; Kallingal and Mintz, 2014) and *Per1* expression (Gamble et al., 2007) in the SCN and induces phase shifts in behavioral rhythms (Albers et al., 1995; Gamble et al., 2007; Kallingal and Mintz, 2007, 2010; Sterniczuk et al., 2014) that are enhanced when coadministered with vasoactive intestinal peptide (VIP; Albers et al., 1995). SCN GRPNs show increased c-Fos expression after light exposure (Earnest et al., 1993), suggesting their involvement in conveying photic information to the SCN for entrainment. However, *Grpr* knock-out mice display attenuated phase shifts in response to high-lux light pulses, but not to low-lux light pulses (Aida et al., 2002), suggesting that GRP plays a partial but nonessential role in photic entrainment. Discrepancies between GRP administration and *Grpr* knock-out phenotypes may reflect the limitations of exogenous GRP in accurately mimicking physiological GRP action, leaving the precise role of GRP signaling in photic entrainment open to debate.

The involvement of GRP signaling in the generation and maintenance of circadian rhythms remains unclear. Mice lacking GRPR exhibited normal behavioral rhythms under standard light-dark cycles and constant darkness (Aida et al., 2002). However, GRP signaling may be important for maintaining neural firing rhythms in the absence of VIP signaling (Brown et al., 2005), suggesting a compensatory mechanism under certain conditions. The limited specificity of experimental tools targeting distinct neuronal populations has restricted the study of GRPNs in circadian clock functions. Consequently, their role in generating circadian rhythms and their precise mechanisms contributing to photic entrainment remain largely unexplored. Resolving these issues will deepen our understanding of the cellular and molecular mechanisms underlying circadian rhythm regulation and its synchronization with environmental light cues.

The functional complexity of GRPNs extends beyond GRP signaling. GRPNs are GABAergic and coexpress VIP (Moore, 2013; Wen et al., 2020), implicating them in multiple signaling pathways within the SCN network. This multifaceted nature of GRPNs cannot be fully captured by simply knocking out *Grp* or *Grpr*. A nuanced approach that considers their diverse molecular and functional properties is required to elucidate their contribution to circadian rhythms. Recent advancements in genetic tools have enabled selective manipulation of specific neuronal populations, facilitating the dissection of their roles in complex circuits. *Grp-iCre* knock-in (KI) mouse line has been instrumental in studying GRPNs, allowing the targeted expression of genes within these neurons (Inoue et al., 2018). This model is particularly advantageous for exploring the functions of GRPNs.

In this study, we used *Grp-iCre* KI mice to explore the specific roles of SCN GRPNs in circadian rhythm generation and photic entrainment. By employing circuit tracing, calcium imaging, and behavioral assays, we characterized neuronal outputs and inputs, as well as the functional significance of GRPNs within the SCN. Our findings provide new insights into the contributions of these neurons to circadian biology and highlight their crucial roles in mediating photic entrainment. These results establish GRPNs as integral components of the SCN and underscore their multifaceted roles in maintaining circadian rhythmicity and photic entrainment. This study lays the groundwork for future research on the interactions between GRPNs and other SCN neuronal populations in orchestrating the complex processes underlying circadian regulation.

## Materials and Methods

### Animals

*Grp-iCre* KI mice have been described previously (Inoue et al., 2018). *As:mPer2^Luc^* (referred to as PER2::LUC) mice were obtained from the Jackson Laboratory (stock no. 006852). *Grp-iCre* KI homozygotes with a C57BL6/J background were used for behavioral assessment of *Grp* knock-out mice; for the other experiments, *Grp-iCre* KI heterozygotes were used. Ten- to 20-week-old male mice were used in this study. The mice were maintained under a 12 h light/dark cycle (LD) or constant darkness (DD) in a temperature- and humidity-controlled room, with food and water provided *ad libitum*. All experimental procedures were approved by the Animal Experiment and Use Committee of the University of Tsukuba and were performed in accordance with the NIH guidelines.

### Activity

The animal activity was measured using infrared light sensors or running wheels. The mice were housed individually in their home cages with infrared light sensors (Lab Design, catalog #ACT-1) or in cages equipped with running wheels (Melquest, catalog #RWC-15). The cages were maintained in light-tight chambers illuminated with white light-emitting diode (LED; 250 lux). Both infrared light locomotor and wheel-running activities were recorded in 1 min bins (ClockLab, Actimetrics) in either LD or DD. Data obtained from 14 d each of LD and DD were used for analysis.

To measure the phase shift by light pulse, mice were kept under 12 h LD condition for at least 7 d and were exposed to a 30 min white LED light pulse of 250 lux at either zeitgeber (ZT) 14 or ZT22 and switched to complete darkness. Their activity in the DD after light stimulation was measured for at least a week. To measure the phase shift from a 6 h phase advance or delay, mice were kept in 12 h LD condition for at least 7 d and then subjected to 6 h of phase advance or delay. After the induction of jet lag, the mice were kept in the new phase for at least 10 d to assess re-entrainment.

The onset and offset of wheel-running activity in each mouse were determined using ClockLab Analysis 6 (Actimetrics), and the time point with the largest difference in activity level 6 h before and after the point was determined to be the onset and offset (when after was greater than before, it was considered the onset; when before was greater than after, it was considered the offset). The 50% phase shift value (PS50) was calculated by fitting the onsets (for phase advances) or offsets (for phase delays) for 14 d (4 d before and 10 d after the jet lag) to a sigmoidal dose–response curve with a variable slope, *Y* = Bottom + (Top − Bottom) / (1 + 10^(log PS50 − *X*) Hillslope^), where *X* is the log of the dose, *Y* is the response, Top and Bottom are the plateaus of *Y*, and Hillslope is the slope factor. Data that did not fit the sigmoidal dose–response curve (negative *R* squared value) were excluded. Negative PS50 values were also excluded. The circadian period and Qp value (the power of the chi-square periodogram used as an indicator of rhythm robustness) were calculated using ClockLab Analysis 6.

### Virus

*pcDNASADB19L* (#32632), *pcDNA-SADB19G* (#32633), *pcDNA-SADB19N* (#32630), *pcDNA-SADB19P* (#32631), *pSADdeltaG-GFP-F2* (#32635), *pAAV-EF1a-DIO-hM3D(Gq)-mCherry* (#50460), *pAAV-EF1a-DIO-mCherry* (#50462), *pAAV-flex-taCasp3-TEVp* (#45580), *pAAV-EF1a-double floxed-hChR2(H134R)-EYFP-WPRE-HGHpA* (#20298), and *pAAV-Ef1a-DIO-GCaMP6s*, *pAAV-Ef1a-DIO-GFP* were obtained from Addgene. *pAAV-CAG-FLEX(Frt)-TVA-mCherry* and *pAAV-CAG-FLEX(Frt)-RG* (rabies glycoprotein; RG) were provided by Naoshige Uchida (Harvard University). *pAAV-EF1a-NLS-tdTomato* was created by replacing the sequence encoding floxed hM3D(Gq)-mCherry in *pAAV-EF1a-DIO-hM3D(Gq)-mCherry* with that encoding nuclear localization signals (NLS) and tdTomato using the In-Fusion HD Cloning Kit (CloneTech, catalog #639648). The backbone vector and tdTomato coding sequences were amplified using CloneAmp HiFi PCR Premix (CloneTech, catalog #639298).

*SADΔG-GFP(EnvA)* was generated by transfecting *pcDNA-SADB19L*, *pcDNA-SADB19G*, *pcDNA-SADB19N*, *pcDNA-SADB19P*, and *pSADdeltaG-GFP-F2* into B7GG cells, followed by pseudotyping in BHK-RGCD-EnvA cells and ultracentrifugation (Osakada and Callaway, 2013; Saito et al., 2018). AAVs were generated using the AAVpro Helper Free System (catalog #6232; Takara). pHelper, pRC, and each pAAV vector were transfected into HEK293T cells using polyethylenimine (Polysciences, #24765) according to a standard protocol. Three days after transfection, the virus was purified using an AAV extraction solution (catalog #6235; Takara) according to the manufacturer’s protocol. The titers of recombinant virus vectors were as follows: *SADΔG-GFP(EnvA)*, 1.2 × 10^9^ (infectious unit/ml). The titer of AAV vectors (genomic copies/ml) was determined by real-time PCR using primer sets for the WPRE sequences. *AAV2-EF1a-DIO-hM3D(Gq)-mCherry*; 1.0 × 10^13^ gc/ml, *AAV2-EF1a-DIO-mCherry*; 1.0 × 10^13^ gc/ml, *AAV10-flex-taCasp3-TEVp*; 5.0 × 10^12^ gc/ml, *AAV2-Ef1a-DIO-hChR2(H134R)-EYFP*; 1.4 × 10^13^ gc/ml, *AAV2-Ef1a-DIO-GCaMP6s*; 5.0 × 10^12^ gc/ml, *AAV2-Ef1a-DIO-GFP*; 1.0-2.5 × 10^13^ gc/ml, *AAV2-NLS-EF1a-tdTomato*; 1.0 × 10^13^ gc/ml *AAV-CAG-FLEX(Frt)-TVA-mCherry*; 3.74 × 10^12^ gc/ml, *AAV-CAG-FLEX(Frt)-RG*; 1.91 × 10^11^ gc/ml.

### Surgery

The mice were deeply anesthetized with 2% isoflurane inhalation solution (Viatris) for all procedures. They were positioned within a stereotaxic frame (David Kopf Instruments). The coordinates of injection for the SCN were as follows: AP, −0.35 mm; ML, ±0.2 mm from bregma; DV, −5.8 mm from the skull surface. The injection volume at each site was 132 nl with a glass microcapillary syringe using an air pressure injector system (Picospritzer III, Parker). We waited 5 min before removing the syringe after injection.

To determine Cre expression in the GRPNs, *AAV2-EF1a-DIO-mCherry* was bilaterally injected into the SCN of *Grp-iCre* KI mice. For the anterograde tracing of SCN GRPNs, *AAV2-Ef1a-DIO-ChR2-EYFP* was bilaterally injected into the SCN of *Grp-iCre* KI mice. The mice were housed in their home cages for 2 weeks before perfusion.

For retrograde tracing experiments, a cocktail of *AAV2-Ef1a-DIO-TVA-mCherry* and *AAV2-CAG-DIO-RG* (132 nl, mixed at a 1:2 ratio) was bilaterally injected into the SCN; 2 weeks later, *SADΔG-GFP(EnvA)* was bilaterally injected into the same region. The mice were housed in their home cages for 5 d before perfusion.

*AAV10-Ef1a-DIO-taCasp3-TEVp* mixed with *AAV2-Ef1a-DIO-GFP* and *AAV2-Ef1a-NLS-TdTomato* as markers of the injection site were injected into the SCN of *Grp-iCre* KI mice at a ratio of 3:1:1 to ablate GRP-producing neurons (GRPNx). For control mice, the same ratio of saline was used instead of *AAV10-Ef1a-DIO-taCasp3-TEVp*. For the experiment confirming the efficiency of Caspase-3 (Fig. 4), *AAV10-Ef1a-DIO-taCasp3-TEVp* was mixed with *AAV2-Ef1a-NLS-TdTomato* at a 3:1 ratio, while the same amount of saline was mixed with *AAV2-Ef1a-NLS-TdTomato* for the control group.

For confirming the expression of GRP, arginine vasopressin (AVP), and VIP in caspase-3-induced ablation, we intracerebroventricularly injected 2 μl of 5 μg/μl colchicine (FUJIFILM Wako Chemical Corporation, catalog#035-03853) in each mouse, with the coordinates of AP, +1.00 mm; ML, +0.70 mm from bregma; DV, −3.00 mm from the skull surface. Brain sampling was performed the next day after the colchicine injection.

### Immunohistochemistry

Animals were deeply anesthetized with avertin (2% 2,2,2-tribromoethanol [Sigma-Aldrich, catalog #T48402], 1.2% 2-methyl-2-butanol [FUJIFILM Wako Pure Chemical Corporation, catalog #010-03703], 8% ethanol [FUJIFILM Wako Pure Chemical Corporation, catalog #057-00456]/pH 7.4 phosphate-buffered saline (PBS; Nacalai Tesque, catalog #27575-31)]) and then perfused transcardially with PBS, followed by 4% paraformaldehyde (PFA, Nacalai Tesque, catalog #02890-45) in PBS. Brains were removed and postfixed overnight in 4% PFA at 4°C and transferred to 20% sucrose (FUJIFILM Wako Pure Chemical Corporation, catalog #196-00015) in PBS at 4°C. After overnight incubation, the brains were frozen in liquid nitrogen in the gas phase. Coronal brain sections were sliced at 25 µm using a cryostat (Leica Biosystems).

The serial brain sections were collected in PBS in 12-well plates and incubated in 24-well plates with blocking buffer [0.2 v/v% Triton X-100 (MP Biomedicals, catalog #807426), 3% bovine albumin serum pH 5.2, (Nacalai Tesque, catalog #01863-48)/PBS] for 30 min at room temperature. The sections were incubated with primary antibody in the blocking buffer at 4°C overnight. The sections were rinsed two times for 10 min each with PBS and once for 10 min with blocking buffer followed by secondary antibody treatment at either room temperature for 3 h or 4°C overnight. Sections were counterstained with 1 mg•ml^−1^ cellstain DAPI solution (Dojindo, catalog #D523) diluted by PBS. After incubation, the sections were rinsed three times for 10 min each with PBS, mounted, and coverslipped. The primary antibodies used were rat anti-green fluorescent protein (1:1,000; Nacalai Tesque, catalog #04404-84, RRID: AB_10013361), goat anti-mCherry (1:1,000; SICGEN, catalog #AB0040-200, RRID: AB_2333092), rabbit anti-gastrin-releasing peptide (1:300; Immunostar, catalog #20073, RRID: AB_572221), rabbit anti-vasopressin (1:300; Immunostar, catalog #20069, RRID: AB_572219), and rabbit anti-vasoactive intestinal peptide (1:300; Immunostar, catalog #20077, RRID: AB_572270). The secondary antibodies were as follows: Alexa Fluor 488 donkey anti-rat IgG (1:1,000; Thermo Fisher Scientific, catalog #A21208, RRID: AB_2535794), Alexa Fluor 488 donkey anti-rabbit IgG (1:1,000; Thermo Fisher Scientific, catalog #A21206, RRID: AB_2535792), Alexa Fluor 594 donkey anti-goat IgG (1:1,000; Thermo Fisher Scientific, catalog #A11058, RRID: AB_2534105), Alexa Fluor 594 donkey anti-rabbit IgG (1:1,000; Thermo Fisher Scientific, catalog #A21207, RRID: AB_141637), and Alexa Fluor 647 donkey anti-rabbit IgG (1:1,000; Thermo Fisher Scientific, catalog #A31573, RRID: AB_2536183). Images were captured under a microscope (Leica SP8).

### Chemogenetic manipulation of GRPN

To measure the phase shift by chemogenetic manipulation, *AAV2-Ef1a-DIO-hM3Dq-mCherry* or *AAV2-Ef1a-DIO-mCherry* (control) was injected into the SCN of heterozygous *Grp-iCre* KI mice (GRPN-hM3Dq mice or GRPN-mCherry mice). After recovery from surgery, the mice were housed individually in cages equipped with a running wheel at 250 lux. The mice were entrained to 12 h light/dark (LD) cycle for at least 10 d and were injected with 1 mg•kg^−1^ of clozapine *N*-oxide (CNO; Abcam, catalog #ab141704) or the same volume of saline as control intraperitoneally at either ZT14 or ZT22 and transferred to constant darkness. DD activity was measured for at least a week.

### Fiber photometry

*AAV10-Ef1a-DIO-GCaMP6s* was injected into the SCN of *Grp-iCre* KI mice for Ca^2+^ recording of the GRPN using fiber photometry. For control mice, *AAV2-Ef1a-DIO-GFP* was used. For cannula implant for fiber photometry, Ø0.2 mm fiber optic cannulae with black Ø1.25 mm ceramic ferrule, 200 μm, 0.50 NA (catalog #R-FOC-BL200C-NA, RWD Life Science) were implanted above the SCN with the coordinates: AP, −0.50 mm; ML, +0.2 mm from bregma; DV, −5.6 mm from skull surface. Ca^2+^ signals were detected using a Doric fiber photometry system (Doric Lenses) with a 465 nm light-emitting diode (LED; CLED 465 nm, Doric Lenses) for fluorophore excitation. The emission signal from the 465 nm excitation was collected via a patch cable (Thorlabs) connected to photodetectors (four-port fluorescence mini cube, Doric Lenses) at a sampling rate of 11 kHz. The LED power was adjusted at the tip of the optical fiber to around 20 μW. The values of fluorescence change (*dF/F*) were derived by calculating (*F_t_* − *F_0_*)/*F_0_*, where *F_0_* is the baseline fluorescence signal calculated with the least mean square fit of the whole data series in each session. Experiments were performed at approximately ZT12–13. Each recording was 15 s long, and the mice received 250 lux white light exposure during the middle 5 s. Each mouse received 10 repeated 15 s recordings.

### PER2 bioluminescence recording

The mice were killed by spinal dislocation at ZT5-10. The brains were rapidly removed and placed in 1× Hanks' balanced salt solution (Invitrogen Life Technologies Corporation, catalog #14025-092) on ice. Then, 150 μm coronal sections including the SCN were made with a vibratome (VT1200; Leica Biosystems). Two SCN slices were collected from each mouse, and a small triangular section of ∼2 mm^2^ including the SCN was cut from each slice. The slice is then placed on Millicell Cell Culture Inserts (Merck KGaA; catalog #PICM0RG50) in a 35 mm tissue culture dish (IWAKI AGC Techno Glass; catalog #3000–035) with 1.5 ml of low glucose Dulbecco's modified Eagle's medium (Merck KGaA; catalog #D2902) containing 10 mM HEPES, pH 7.0, 3.5 g L^−1^ d-glucose, 0.1 mM d-luciferin potassium salt (FUJIFILM Wako Pure Chemical Corporation; catalog #126–05116), 2% B-27 supplement (Merck; catalog #17504–044), 35 mg L^−1^ NaHCO_3_ (FUJIFILM Wako Pure Chemical Corporation; catalog #197–01302), and 1% penicillin–streptomycin (10,000 units ml^−1^ penicillin and 10,000 mg ml^−1^ streptomycin; Merck; catalog #15140–122). Cultures were maintained at 37°C, and bioluminescence was recorded for 1 min duration over 7 d with luminometer Kronos Dio (AB-2550, ATTO). Detrended data for 7 d were used for the analysis. The half-life of the amplitude is defined as the time at which the amplitude is less than half of the initial value. Images were obtained using a Zeiss LSM 800 microscope.

### Statistics

No statistical methods were used to determine the sample size. The experiments were randomized, and the investigators were not blinded to the allocation during the experiments or outcome assessment. All analyses used the unpaired Student's *t* test, except for comparison of the Qp value and activity period between the GRPNx mice and control mice (two-way ANOVA) and comparison of the phase shift from saline or CNO intraperitoneal injection for GRPN-hM3Dq and GRPN-mCherry (paired Student's *t* test). Mean ± SEM and individual values are used to show the results. The Qp values and periods were calculated using χ-square periodogram with the ClockLab analysis.

All statistical analyses were performed using GraphPad Prism 10. Differences were considered significant at *p < 0.05, **p < 0.01, and ***p < 0.001.

## Results

### *Grp-iCre* KI mice express iCre recombinase specifically in SCN GRP neurons

**Figure 1.**
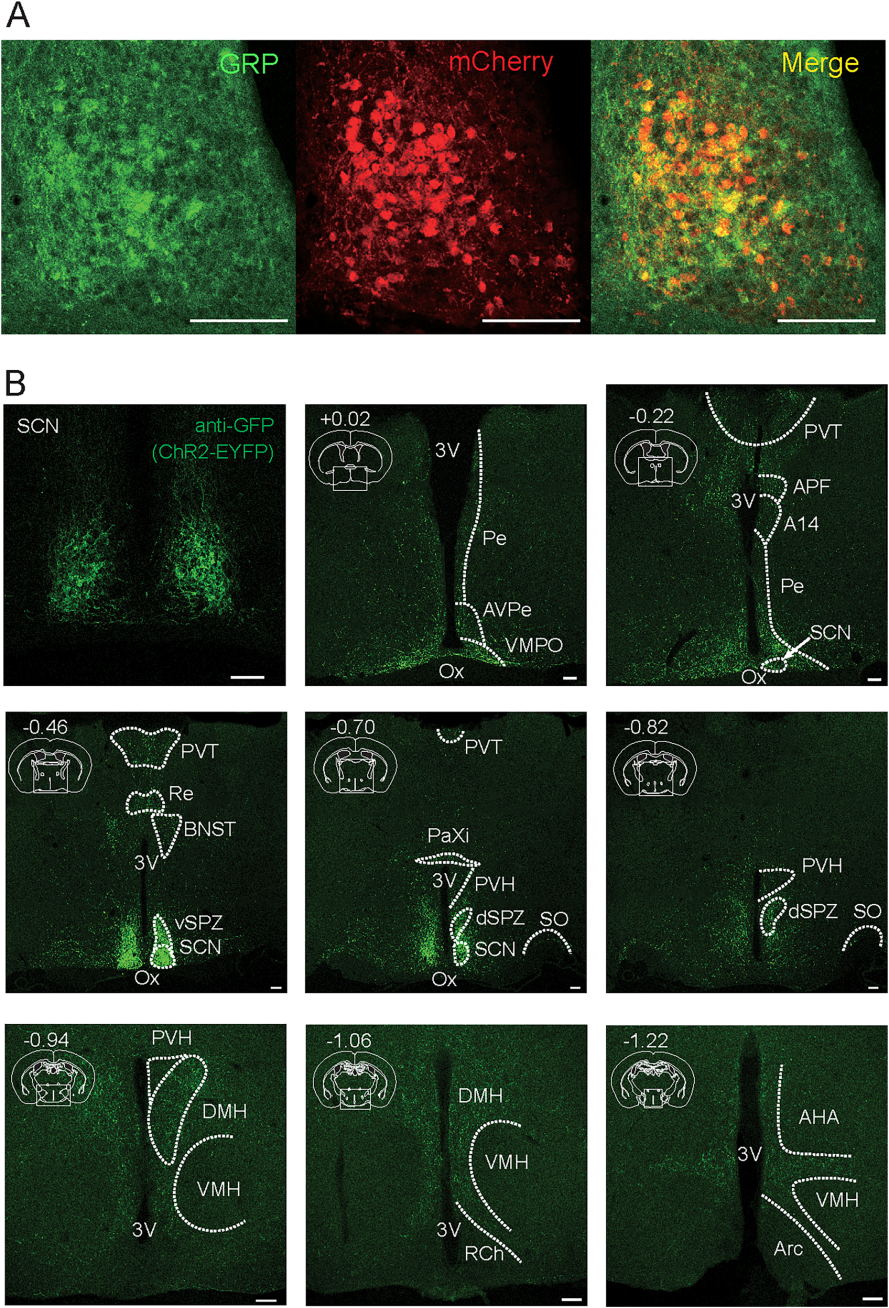
Representative images confirming Cre expression in the SCN and anterograde tracing of SCN GRPNs. ***A***, Representative images of SCN collected from *Grp-iCre* KI heterozygous mouse injected with *AAV2-EF1a-DIO-mCherry* in the SCN. Left image displays endogenous GRP expression visualized by anti-GRP antibody in green fluorescence, middle image displays mCherry as a Cre recombinase activity marker, and right image displays the merged image of both endogenous GRP and mCherry. ***B***, Anterograde tracing of SCN GRPNs expressing ChR2-EYFP. Representative images of axons of SCN GRPNs shown as green fluorescence in the coronal mouse brain sections. White dotted line represents the approximate perimeter of each brain region. On the top left corner of each image is the approximate anterior-posterior axis of each slice. 3V, third ventricle. Pe, periventricular hypothalamic nucleus. AVPe, anteroventral periventricular nucleus. VMPO, ventromedial preoptic nucleus. Ox, optic chiasm. PVT, paraventricular thalamic nucleus. APF, anterior perifornical nucleus. A14, A14 dopamine cells. SCN, suprachiasmatic nucleus. Re, reuniens thalamic nucleus. BNST, bed nucleus of the stria terminalis. vSPZ, ventral subparaventricular zone. PaXi, paraxiphoid nucleus of thalamus. PVH, paraventricular hypothalamic nucleus. dSPZ, dorsal subparaventricular zone of the hypothalamus. SO, supraoptic nucleus. DMH, dorsomedial hypothalamic nucleus. VMH, ventromedial hypothalamic nucleus. RCh, retrochiasmatic area. AHA, anterior hypothalamic area. Arc, arcuate hypothalamic nucleus. All scale bars represent 100 μm.

To specifically manipulate SCN GRPNs, we used *Grp-iCre* KI mice, a previously established mouse line (Inoue et al., 2018) in which the endogenous *Grp* coding sequence was replaced with that of iCre recombinase. Because the efficiency and specificity of iCre recombinase activity in the SCN of *Grp-iCre* KI mice have not been previously characterized, we injected a Cre-dependent AAV carrying mCherry into the SCN of these mice to validate its restricted expression. Immunohistochemical analysis revealed the colocalization of mCherry and endogenous GRP (Fig. 1*A*). mCherry-positive neurons were primarily localized in the center of the SCN, and the majority of mCherry-positive neurons (87 ± 3.6%, *n* = 3) expressed GRP, indicating that iCre recombinase was specifically and efficiently expressed in GRP-positive neurons in the SCN (Fig. 1*A*).

### SCN GRPNs project to multiple nuclei mostly in the thalamus and hypothalamus

To understand the neural circuit through which SCN GRPNs contribute to circadian regulation, we examined both the outputs and inputs of these neurons using anterograde and retrograde neuronal tracing. The Cre-dependent expression of enhanced yellow fluorescent protein (EYFP) fused with channel rhodopsin 2 (ChR2) was used to visualize the axonal projections of SCN GRPNs (Fig. 1*B*). We observed that SCN GRPNs project to multiple nuclei, including the ventromedial preoptic nucleus (VMPO), periventricular hypothalamic nucleus (Pe), anteroventral periventricular nucleus (AVPe), paraventricular thalamic nucleus (PVT), anterior perifornical nucleus (APF), A14 dopamine cells, reuniens thalamic nucleus (Re), bed nucleus of the stria terminalis (BNST), ventral subparaventricular zone (vSPZ), paraxiphoid nucleus of thalamus (PaXi), paraventricular hypothalamic nucleus (PVH), dorsal subparaventricular zone (dSPZ), supraoptic nucleus (SO), and dorsomedial hypothalamic nucleus (DMH). SCN GRPNs also had many intra-SCN projections (Fig. 1*B*). Notably, fluorescence signals were barely detected in the ventromedial hypothalamic nucleus (VMH) and arcuate hypothalamic nucleus (Arc), which are known to receive projections from other neurons in the SCN (Watts and Swanson, 1987).

### SCN GRPNs mainly receive input from neurons within the SCN

**Figure 2.**
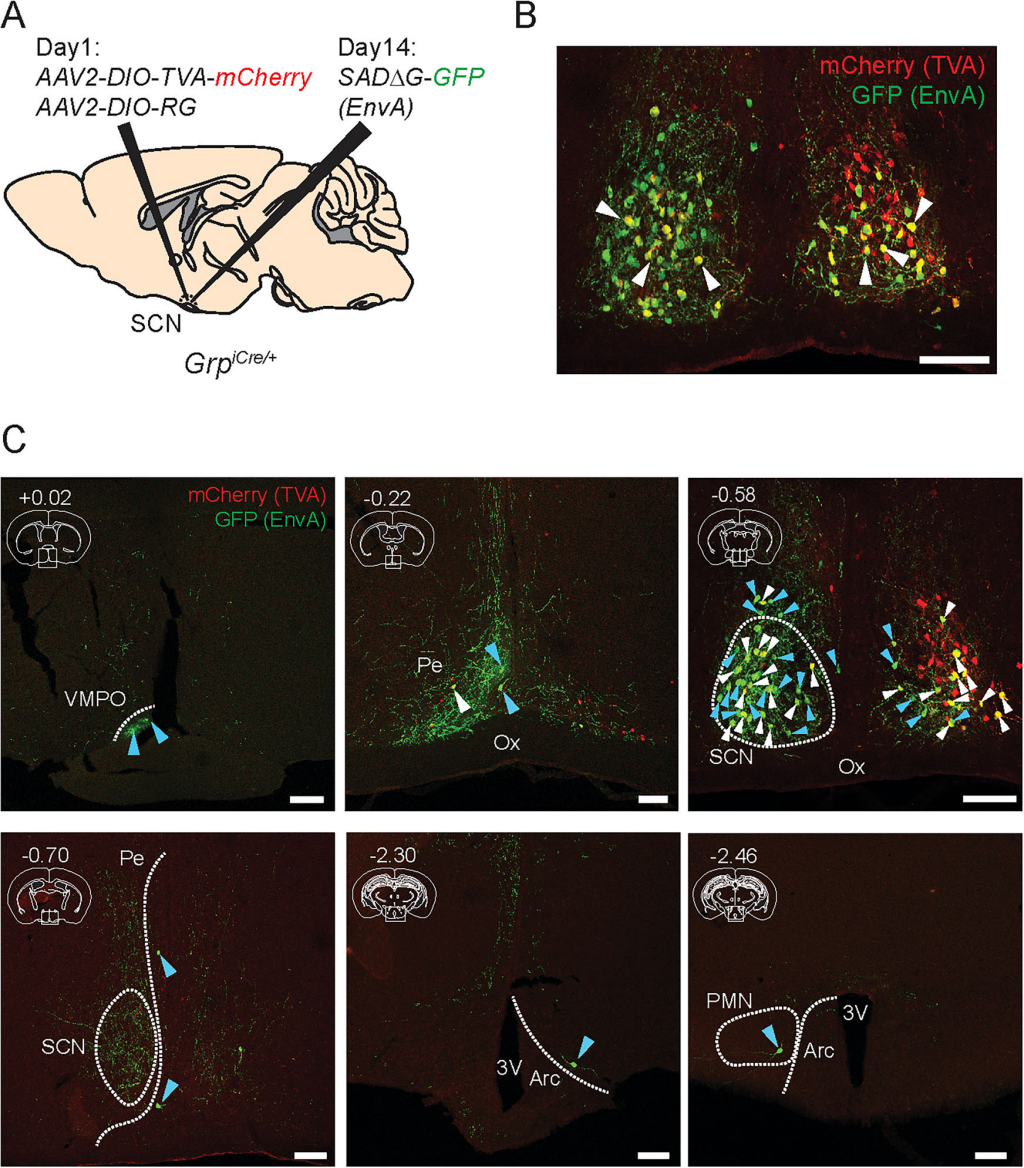
Representative images showing the retrograde tracing of SCN GRPNs. ***A***, Schematic depiction of viral injection in the SCN of *Grp-iCre* KI heterozygous mice for the pseudotyped rabies virus-mediated retrograde tracing. ***B***, Representative image of SCN starter neurons (SCN GRPNs) displaying both TVA-mCherry in red fluorescence and GFP in green fluorescence (infected with rabies virus). ***C***, Representative images displaying the input neurons that project to the SCN GRPNs. TVA-mCherry expression is shown in red fluorescence and GFP is shown as green fluorescence. White dotted line represents the approximate perimeter of each brain region. On the top left corner of each image is the approximate anterior-posterior axis of each slice. 3V, third ventricle. Pe, periventricular hypothalamic nucleus. VMPO, ventromedial preoptic nucleus. Ox, optic chiasm. SCN, suprachiasmatic nucleus. Arc, arcuate hypothalamic nucleus. PMN, premammillary nucleus. White arrow represents starter neurons, which are both mCherry and GFP positive. Blue arrow represents input neurons, which are single positive for GFP. All scale bars represent 100 μm.

We utilized monosynaptic retrograde tracing to identify neurons that form synaptic connections with SCN GRPNs (input neurons). Specifically, we used the pseudotyped rabies vector *SADΔG-GFP(EnvA)* (for details, see Materials and Methods; Fig. 2*A*). Our analysis revealed that most of the neuronal inputs into the SCN GRPNs were found within the SCN (Fig. 2*B*,*C*). Additionally, sparse distribution of input neurons was observed in the hypothalamus, both anterior and posterior to the SCN (Fig. 2*C*). Combined with our anterograde tracing results, these results suggest that SCN GRPNs contribute to SCN output pathways and participate in microcircuit formation within the SCN.

### GRP deficiency does not affect behavioral rhythm or photic entrainment

**Figure 3.**
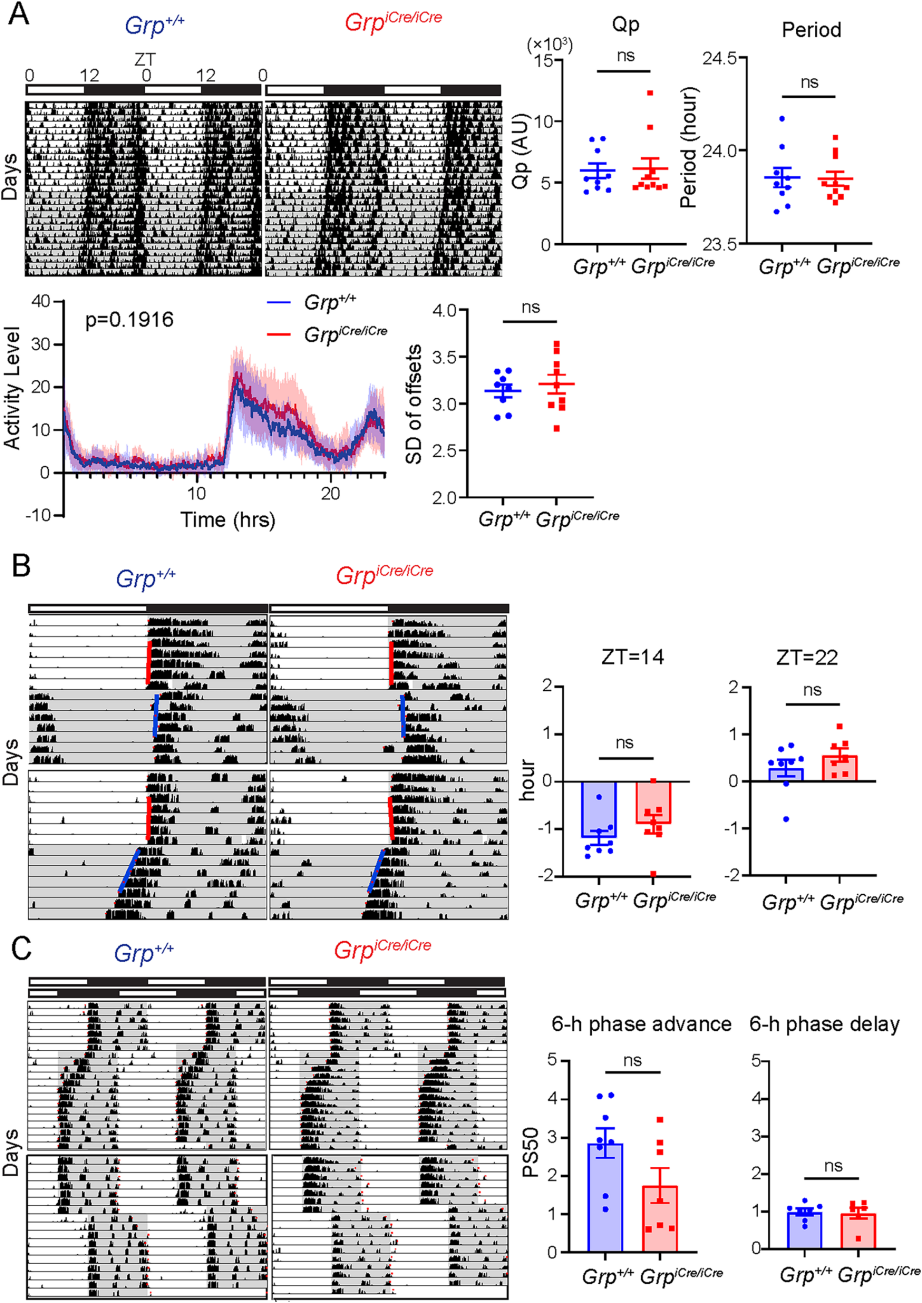
Behavioral rhythm and photic entrainment of GRP KO mice. ***A***, Activity rhythms of wild-type (*Grp^+/+^*) and *Grp-iCre* KI homozygous (*Grp^iCre/iCre^*) mice. Top left depicts actogram of *Grp^+/+^* and *Grp^iCre/iCre^* mice in LD and DD. Bar on top depicts lighting condition and zeitgeber time (ZT). Shaded area represents period where the lights are off. Top right depicts graphs comparing the Qp value and period of each genotype. Bottom left depicts the average activity profile of *Grp^+/+^* and *Grp^iCre/iCre^* mice in LD (*n* = 9 for *Grp^+/+^*, *n* = 10 for *Grp^iCre/iCre^*; *p* = 0.1916, DF = 1439, F = 1.033 for comparison of activity levels at each time point; two-way ANOVA). Bottom right depicts graph comparing the standard deviation of activity offset of *Grp^+/+^* and *Grp^iCre/iCre^* mice in DD (*n* = 8 for *Grp^+/+^*, *n* = 9 for *Grp^iCre/iCre^*; *p* = 0.5731; unpaired Student's *t* test). ***B***, Phase shift of *Grp^+/+^* and *Grp^iCre/iCre^* mice from 30 min light pulse at ZT14 or ZT22. Left depicts actogram of *Grp^+/+^* and *Grp^iCre/iCre^* mice before and after 30 min light pulse. Right depicts graphs comparing the phase shift amount between the two genotypes at ZT14 or 22 (*n* = 8 for *Grp^+/+^* and *Grp^iCre/iCre^* for ZT = 14 light pulse and *n* = 8 for *Grp^+/+^* and *n* = 7 for *Grp^iCre/iCre^* for ZT = 22 light pulse; *p* = 0.2508 for comparison of phase shift amount for ZT = 14 light pulse, *p* = 0.2570 for comparison of phase shift amount for ZT = 22 light pulse; unpaired Student’s *t* test). Red line in actogram indicates the onset fit determined by ClockLab before light pulse, and blue line indicates onset fit after light pulse. ***C***, Re-entrainment to 6 h phase advance or phase delay jet lag of wild-type (*Grp^+/+^*) and *Grp-iCre* KI homozygous (*Grp^iCre/iCre^*) mice. Left depicts actogram of *Grp^+/+^* and *Grp^iCre/iCre^* mice before and after 6 h phase shifts. Right depicts graphs comparing the PS50 of the two genotypes (*n* = 8 for *Grp^+/+^*, *n* = 7 for *Grp^iCre/iCre^*; *p* = 0.0855 for comparison of PS50 for 6 h phase advance, *p* = 0.6864 for comparison of PS50 for 6 h phase delay; unpaired Student’s *t* test).

Many studies have examined the effects of exogenous administration of GRP; however, few have examined the intrinsic role of GRP signaling in the SCN. In *Grp-iCre* KI mice, the coding sequence for *iCre* replaces the endogenous *Grp* coding sequence, leading to a loss of GRP production in homozygous mice (*Grp^iCre/iCre^*; Inoue et al., 2018). We used *Grp^iCre/iCre^* mice to assess the effects of GRP deficiency on locomotor activity rhythms and photic entrainment. Locomotor activity was monitored in *Grp^iCre/iCre^* mice and their littermate controls (*Grp^+/+^*) under LD or DD conditions for at least 2 weeks (Fig. 3*A*). No significant differences were observed in the Qp values or circadian periods in DD between the groups (*n* = 9 for *Grp^+/+^*, *n* = 10 for *Grp^iCre/iCre^*; *p* = 0.8747 for comparison of Qp values, *p* = 0.9344 for comparison of periods; unpaired Student’s *t* test; Fig. 3*A*). Activity profile of these mice in LD displayed no significant difference (*n* = 9 for *Grp^+/+^*, *n* = 10 for *Grp^iCre/iCre^*; *p* = 0.1916, DF = 1,439, *F* = 1.033 for comparison of activity levels at each time point; two-way ANOVA; Fig. 3*A*). The standard deviation of the activity offset of the two groups of mice from day to day in DD is also compared, and no significant difference was observed (*n* = 8 for *Grp^+/+^*, *n* = 9 for *Grp^iCre/iCre^*; *p* = 0.5731; unpaired Student's *t* test; Fig. 3*A*), indicating that the activity pattern between the two groups is comparable. These results are consistent with those of previous studies, which have reported that GRP receptor deficiency does not substantially alter the behavioral phenotype (Aida et al., 2002).

Aida et al. suggested that GRP signaling may play a limited role in photic entrainment in *Grpr* knock-out mice. In this study, we examined the effect of GRP deficiency on the phase shift induced by light pulses. *Grp^+/+^* and *Grp^iCre/iCre^* mice were entrained to the LD cycle for at least 10 d, a 30 min light pulse was given at either ZT14 or 22, and the mice were subsequently subjected to DD conditions (Fig. 3*B*). There were no significant differences between the two genotypes in the amount of phase advance or delay (*n* = 8 for *Grp^+/+^* and *Grp^iCre/iCre^* for ZT = 14 light pulses and *n* = 8 for *Grp^+/+^* and *n* = 7 for *Grp^iCre/iCre^* for ZT = 22 light pulses; *p* = 0.2508 for comparison of the phase shift amount for ZT = 14 light pulses, *p* = 0.2570 for comparison of the phase shift amount for ZT = 22 light pulses; unpaired Student’s *t* test; Fig. 3*B*). We also subjected these mice to a 6 h phase advance and phase delay jet lag. No significant difference was observed in the speed of re-entrainment after a 6 h advance or delay jet lag (*n* = 8 for *Grp^+/+^*, *n* = 7 for *Grp^iCre/iCre^*; *p* = 0.0855 for comparison of PS50 for 6 h phase advance, *p* = 0.6864 for comparison of PS50 for 6 h phase delay; unpaired Student’s *t* test; Fig. 3*C*). These results suggest that *Grp* is not essential for photic entrainment, despite prior evidence suggesting possible limited involvement of its receptor (Aida et al., 2002).

### Effects of ablation of the SCN GRPNs on behavioral and PER2::LUC rhythm

**Figure 4.**
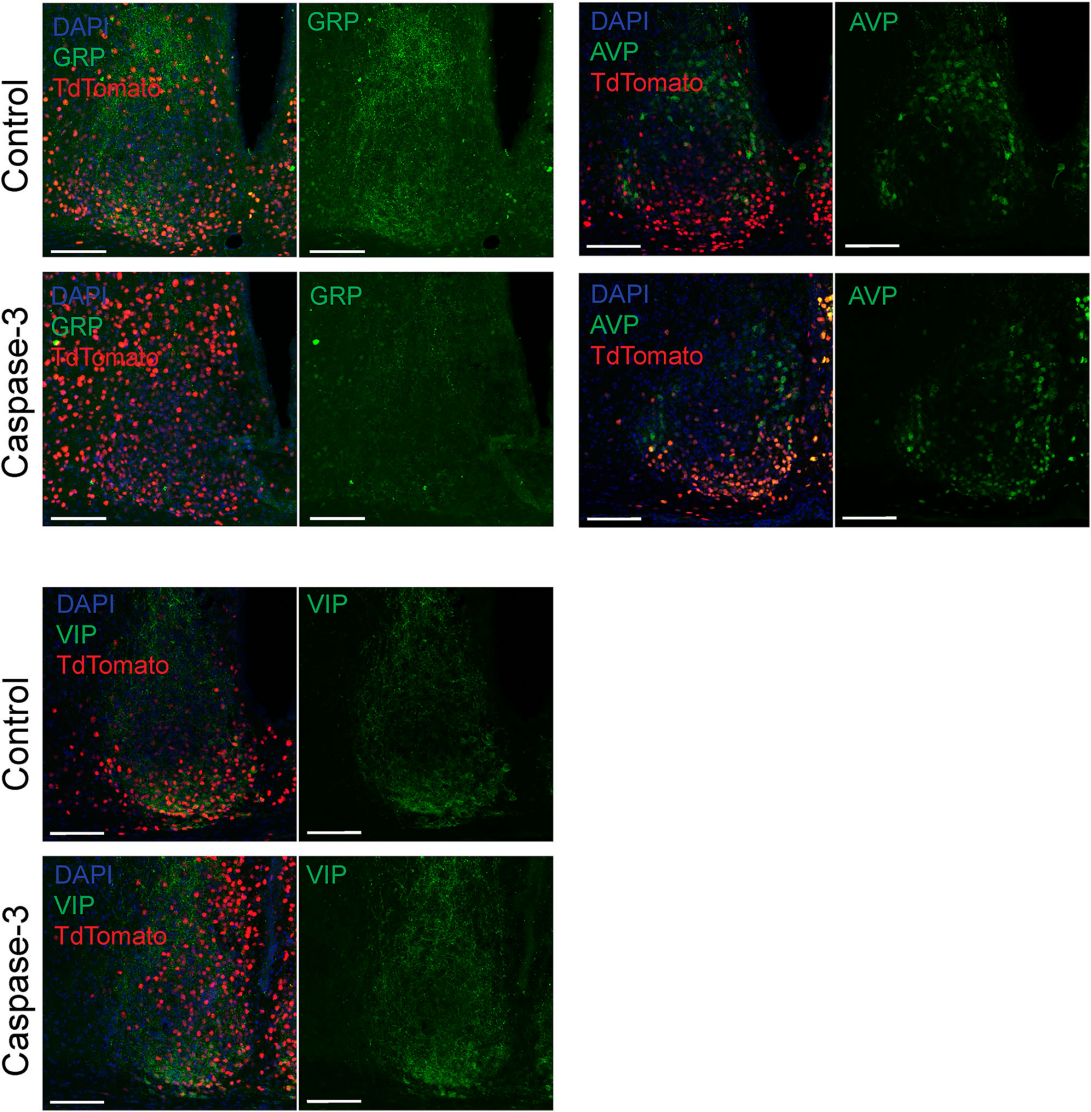
Confirming the effect of caspase-3 in neural ablation. All images show the nuclei stained with DAPI (blue) and TdTomato (red). The four images in the top left display expressions of GRP (green), the right four display AVP (green), and the bottom four display VIP (green). The two left images in each of the four-imaged panel display DAPI staining (blue) and TdTomato (red). The two right images in each of the four-imaged panel display the respective neuropeptide in green. Top two images in each four-imaged panel display the control group that received saline mixed with TdTomato, and the bottom two images in each four-imaged panel display the group receiving caspase-3 and TdTomato virus. Scale bar is 100 μm.

Previous studies demonstrated that SCN GRPNs release various neuromodulators, including VIP and GABA (Moore, 2013; Wen et al., 2020). Consequently, GRPN function is not necessarily mediated solely by the GRP peptide. To investigate the effect of GRPN ablation (GRPNx) on behavioral and molecular rhythms, we performed caspase-3-induced ablation of GRPNs in *Grp-iCre* KI mice. Caspase-3, an enzyme that induces cell death, is specifically expressed in the SCN of these mice. We first confirmed successful ablation of GRP-producing neurons via immunohistochemistry for endogenous GRP. Caspase-3 was coexpressed with a TdTomato reporter in the SCN of *Grp-iCre* KI heterozygous mice, and a reduction of GRP expression was observed compared with control mice that received a saline mixed with TdTomato-expressing virus (*n* = 4 for control group and Caspase-3 group; Fig. 4). Notably, the expression of other neuropeptides, such as VIP and AVP, was unaffected by Caspase-3 expression (*n* = 4 for control group and Caspase-3 group for both VIP and AVP; Fig. 4).

**Figure 5.**
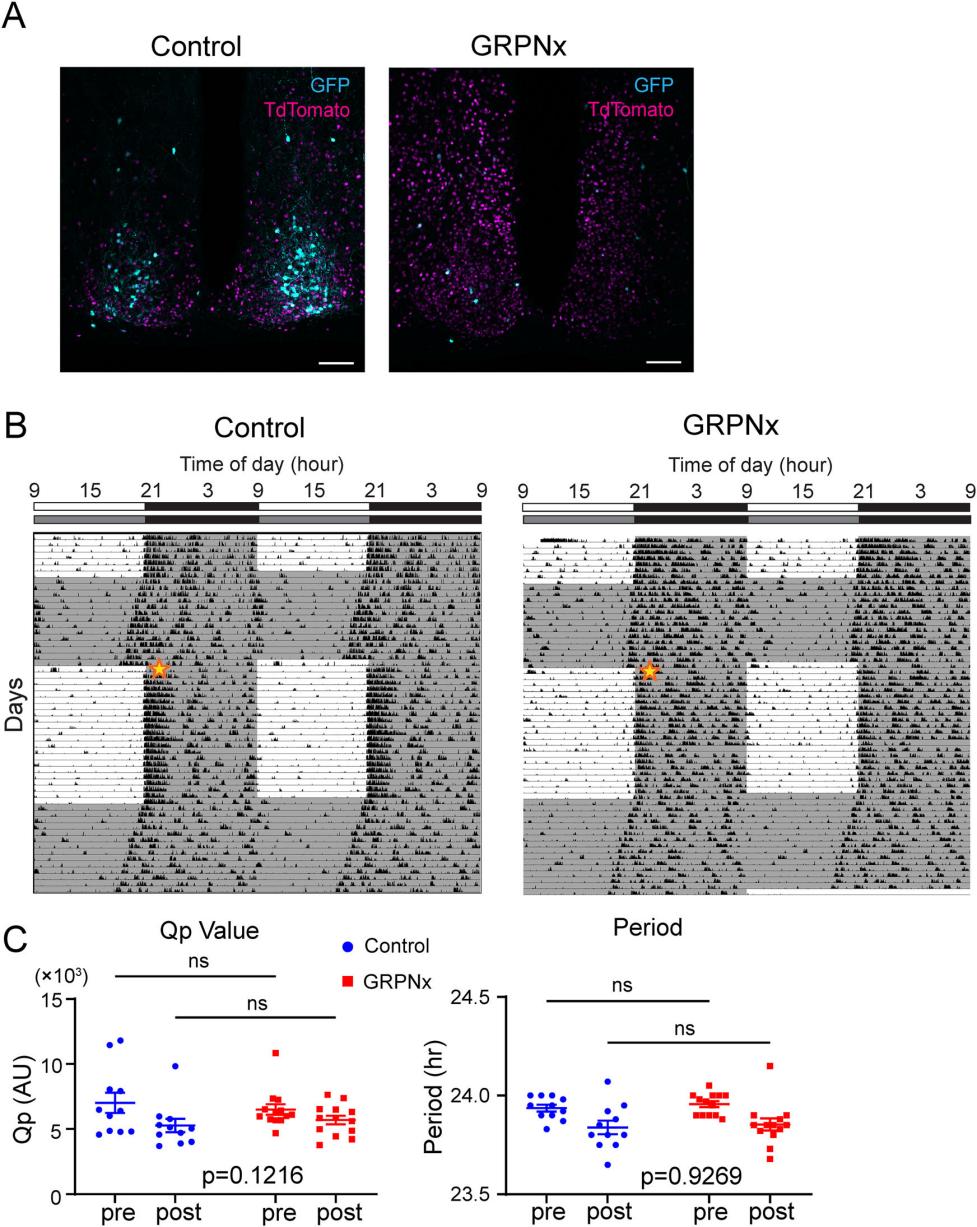
Behavioral rhythm of control and GRPNx mice. ***A***, Representative images of SCN of each group. Left image shows the expression of GFP (cyan) and TdTomato (magenta) in the control group, and right image shows the GRPNx group. GFP (cyan) was used to confirm the cell ablation, while TdTomato (magenta) was used for marker of the injection site. Scale bar is 100 μm. ***B***, Actogram of control and GRPNx group. Bar on top displays ZT and lighting condition. Gray shade in the actogram represents period where the lights were off. Star represents time point of virus injection. Actograms are double plotted. ***C***, Left graph depicts comparison of Qp value between control and GRPNx group. Right graph depicts comparison of period (*n* = 11 for control, *n* = 13 for GRPNx; *p* = 0.1216, DF = 1, *F* = 2.594 for Qp value; *p* = 0.9269, DF = 1, *F* = 0.008605 for period; two-way ANOVA).

For behavioral analysis, the injection site was confirmed by coexpression of TdTomato, while successful ablation was verified by the absence of Cre-dependent GFP expression (Fig. 5*A*). GRPNx mice exhibited a significantly lower number of GFP-positive cells compared with control mice (data not shown). However, no significant differences were observed between GRPNx and control mice in either the Qp value or circadian activity period (*n* = 11 for control, *n* = 13 for GRPNx; *p* = 0.1216, DF = 1, *F* = 2.594 for Qp value, *p* = 0.9269, DF = 1, *F* = 0.008605 for period; two-way ANOVA; Fig. 5*B*,*C*).

**Figure 6.**
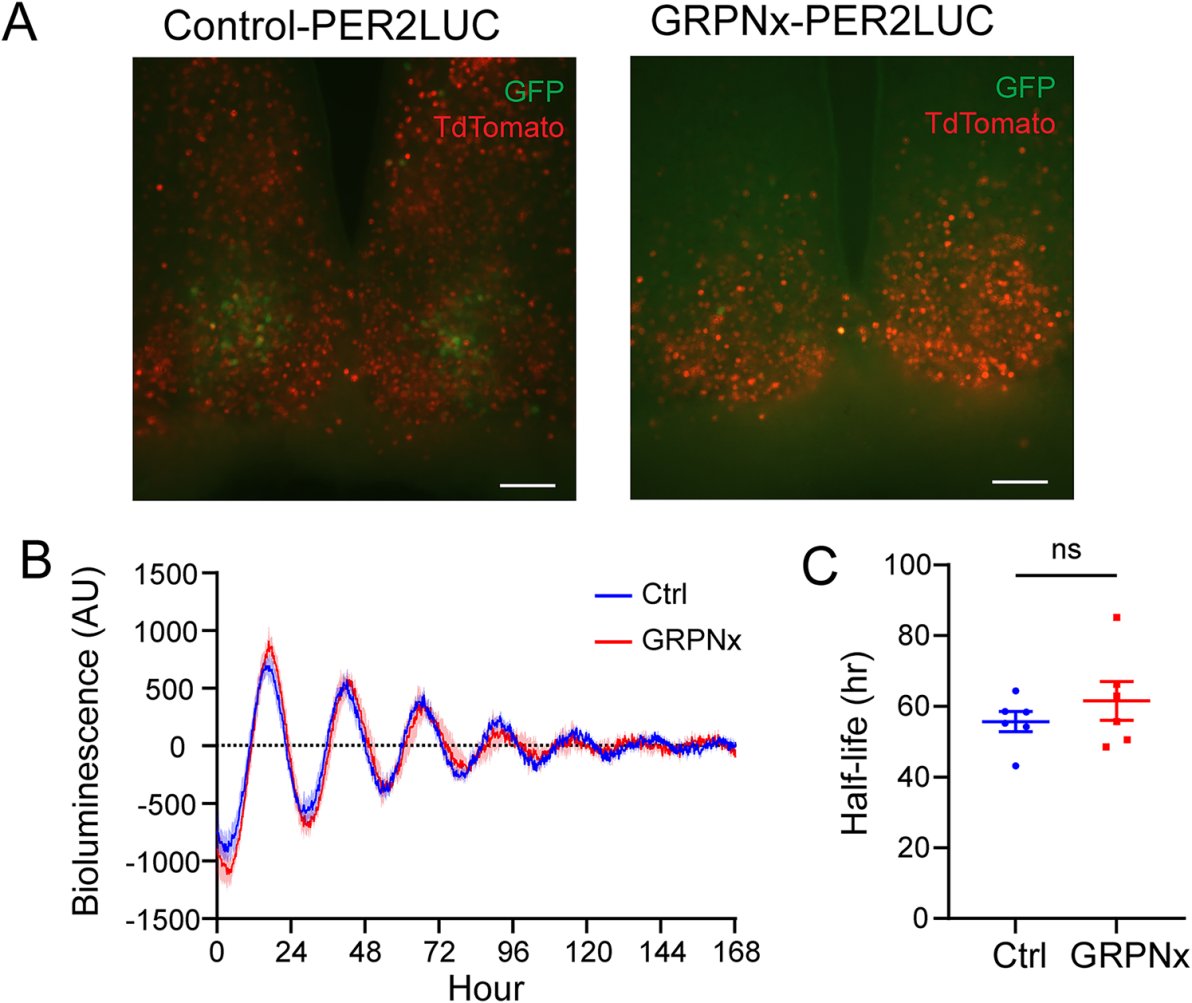
PER2 rhythm of control and GRPNx mice. ***A***, Representative images of SCN slices of each group showing the expression of GFP (green) and TdTomato (red) in *Grp-iCre* KI;*PER2::LUC* mice receiving AAV expressing caspase-3 (GRPNx-PER2LUC) and control (Control-PER2LUC). Scale bar is 100 μm. ***B***, Detrended bioluminescence rhythms of PER2::LUC in the SCN slice cultures prepared from control (blue) and GRPNx (red) mice. ***C***, Comparison of the half-life of the amplitude (damping rate) of PER2::LUC bioluminescence rhythms in control (blue) and GRPNx (red) group (*n* = 6 for both control and GRPNx group; *p* = 0.3666; unpaired Student's *t* test).

We also crossed *Grp-iCre* KI mice with PER2::LUC mice to generate *Grp-iCre* KI;PER2::LUC mice to examine molecular rhythms in the SCN. We ablated SCN GRPNs from these mice using the aforementioned method and monitored PER2 rhythms in SCN slice cultures (Fig. 6*A*,*B*). We found no significant difference in the half-life of the PER2 rhythm between GRPNx mice and control mice (*n* = 6 for both control and GRPNx groups; *p* = 0.3666; unpaired Student's *t* test; Fig. 6*C*). This suggests that SCN GRPNs are not essential for the generation of behavioral rhythms or core clock protein rhythms.

### Possible redundant role of SCN GRPNs in photic entrainment

**Figure 7.**
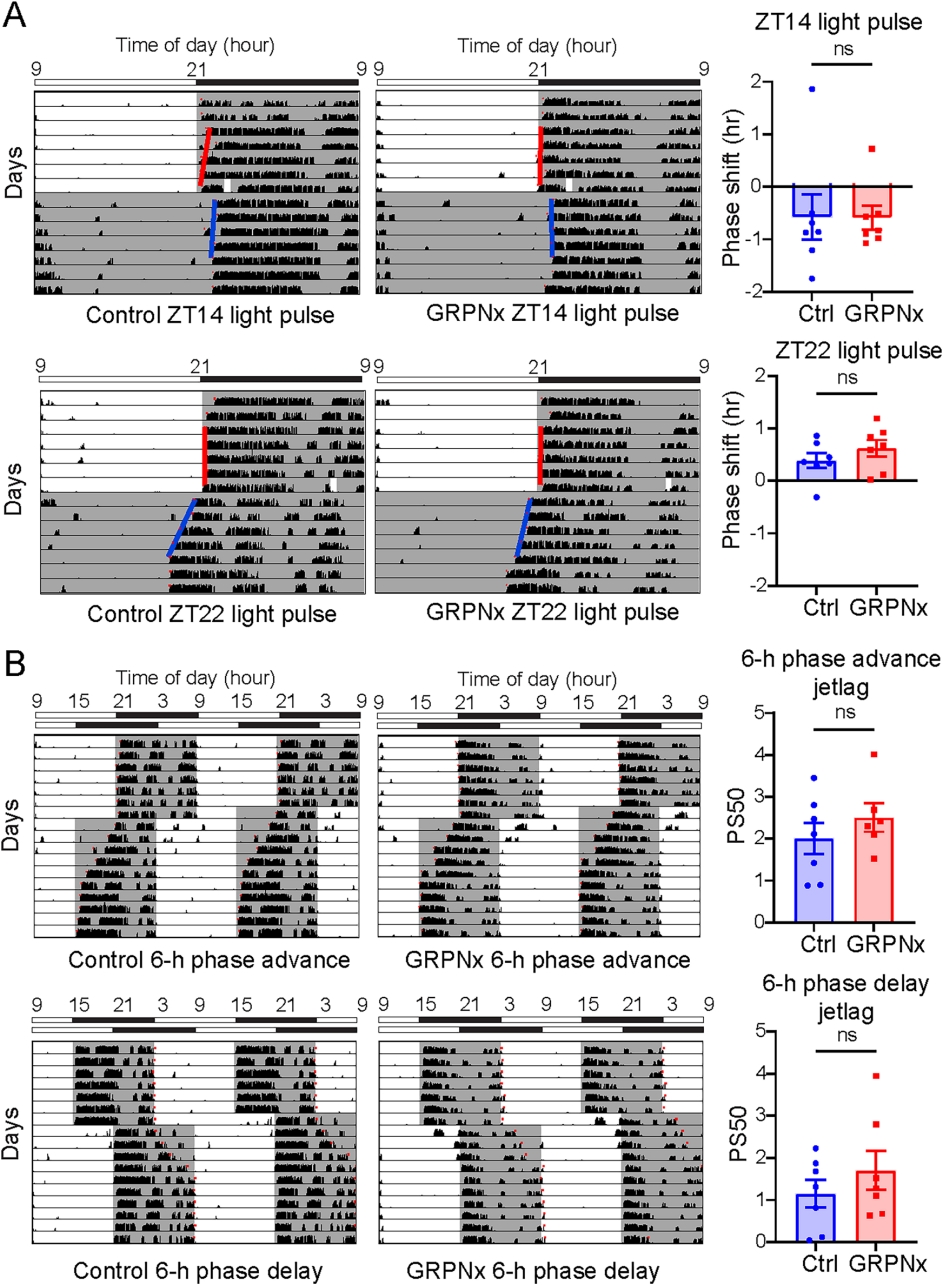
Effect of GRPNx on photic entrainment. ***A***, Left depicts representative actograms of control or GRPNx mice before and after 30 min light pulse at either ZT14 or 22. Bar on top displays time of day and lighting condition. Right depicts graphs comparing the phase shift amount between control and GRPNx group at either ZT14 or 22 (*n* = 7 for both control and GRPNx; *p* = 0.9773 for ZT = 14 light pulse, *p* = 0.2935 for ZT = 22 light pulse; unpaired Student’s *t* test). Red line in actogram indicates the onset fit determined by ClockLab before light pulse, and blue line indicates onset fit after light pulse. ***B***, Left depicts representative actograms of control or GRPNx mice before and after 6 h phase advance or phase delay jet lag. Bar on top displays time of day and lighting condition. Right depicts graphs comparing the PS50 between control and GRPNx group for either 6h phase advance or phase delay jet lag (*n* = 7 for control, *n* = 7 for GRPNx for 6 h phase advance, *n* = 6 for GRPNx for 6 h phase delay; *p* = 0.3468 for comparison of PS50 for 6 h phase advance, *p* = 0.3481 for comparison of PS50 for 6 h phase delay; unpaired Student's *t* test).

Both in vivo and ex vivo administration of GRP has been shown to induce phase shifts in behavioral and core clock gene rhythms (Albers et al., 1995; Gamble et al., 2007; Kallingal and Mintz, 2007, 2010; Sterniczuk et al., 2014). Based on these findings, we investigated whether ablation of SCN GRPNs would affect photic entrainment. Using the same caspase-3 ablation method described above, we ablated GRPNs in the SCN and subjected the mice to light pulses at either ZT14 or ZT22. Our analysis revealed no significant difference in the magnitude of phase shifts between GRPNx and control mice at either time point (*n* = 7 per group; *p* = 0.9773 for ZT = 14 light pulse, *p* = 0.2935 for ZT = 22 light pulse; unpaired Student's *t* test; Fig. 7*A*).

We further assessed photic entrainment by subjecting the mice to 6 h phase advance and phase delay jet lag protocols. Again, no significant differences in re-entrainment speed were observed between GRPNx and control mice for either the phase advance or phase delay condition (*n* = 7 for control, *n* = 7 for GRPNx for 6 h phase advance, *n* = 6 for GRPNx for 6 h phase delay; *p* = 0.3468 for comparison of PS50 for 6 h phase advance, *p* = 0.3481 for comparison of PS50 for 6 h phase delay; unpaired Student's *t* test; Fig. 7*B*). We have also confirmed that there is no correlation between the number of remaining GFP-positive neurons and the strength of the phenotype, suggesting that the lack of significant difference is not due to the compensation from the surviving GRPNs (*p* = 0.5852 for correlation between remaining GFP-positive neurons and phase shift from ZT22 light pulse; *p* = 0.0898 for correlation between remaining GFP-positive neurons and phase shift from ZT14 light pulse; *p* = 0.9164 for correlation between remaining GFP-positive neurons and PS50 from 6 h phase advance; *p* = 0.8163 for correlation between remaining GFP-positive neurons and PS50 from 6 h phase delay; data not shown).

These results suggest that although GRPNs may contribute to phase modulation in response to light, they are not essential for photic entrainment. This implies a functional redundancy within the SCN network, where other neuronal populations, such as VIP-expressing neurons, likely play a more dominant role in mediating photic input to the circadian clock.

### Increase in neural activity in the SCN GRPNs by light exposure

**Figure 8.**
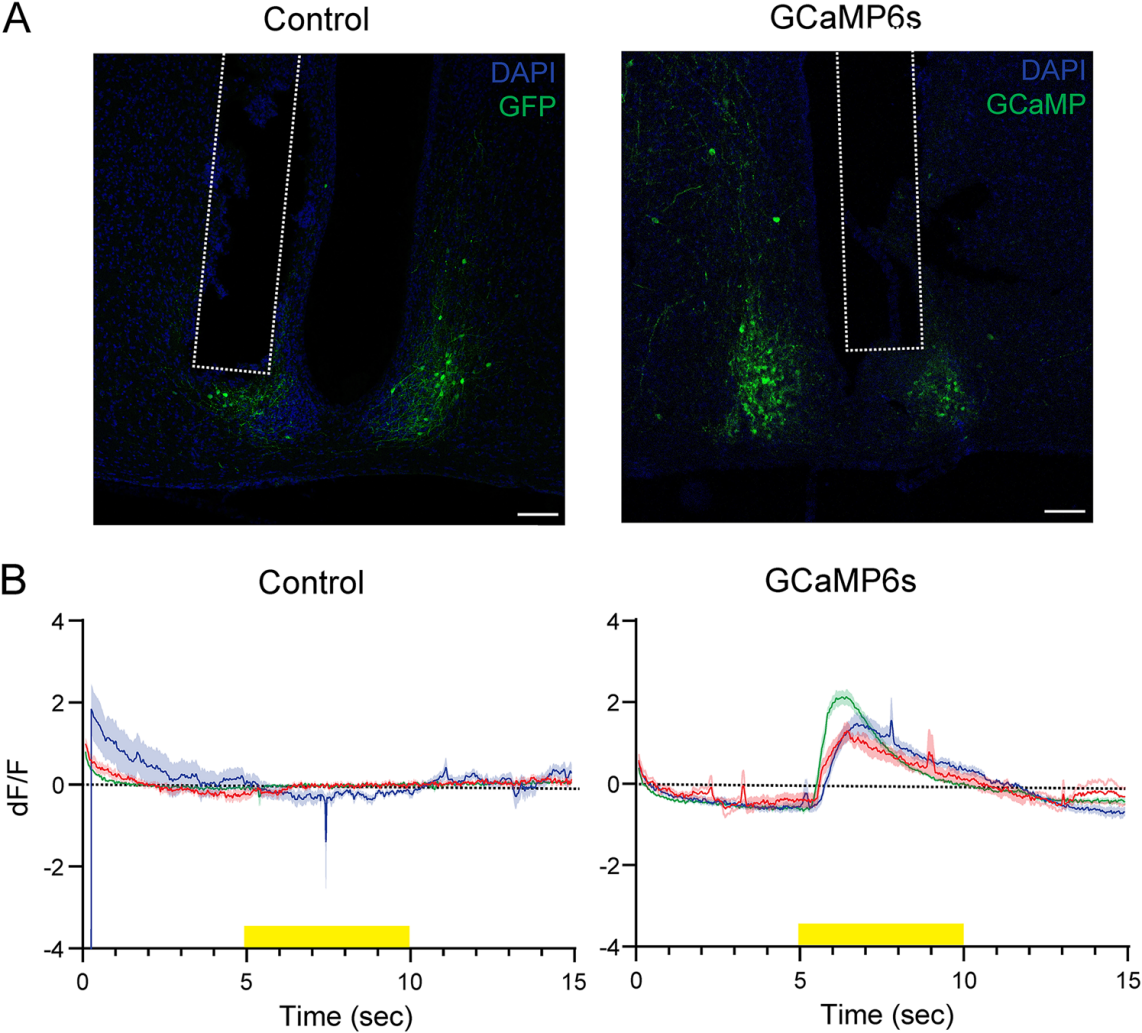
Ca^2+^ dynamics of SCN GRPNs during light exposure to the retina. ***A***, Representative images of SCN of each group showing the location of the optic fiber (white dotted line) and the expression of GCaMP6s (green) and DAPI. Scale bar is 100 μm. ***B***, Ca^2+^ dynamics of SCN GRPNs in the control (GFP) group and GCaMP6s group. Ca^2+^ dynamics was recorded from three mice for each group (10 trials per mouse) and are plotted with the mean ± SEM. Different color represents each individual mouse. The yellow bar on the bottom depicts the time when the mouse is exposed to white light.

Previous studies have shown that the core region of the SCN, where most GRPNs are located, receives direct input from the retina through the retinohypothalamic tract (Moore, 2013). Therefore, we tested whether the SCN GRPNs were activated in response to photic information from the retina. First, we examined the reactivity of SCN GRPNs to light. We expressed the calcium sensor GCaMP6s specifically in GRPNs in the SCN of *Grp-iCre* KI mice and implanted optic fibers above the injection site to record fluorescent signals. The mice were briefly exposed to 250 lux white LED light for 5 s, and the calcium activity of GRPNs during white light exposure was measured (Fig. 8*A*). Compared with control mice, mice expressing GCaMP6s displayed increased fluorescence activity during white light exposure (Fig. 8*B*). This indicates that SCN GRPNs respond quickly to photic stimulation of the retina.

### Stimulating SCN GRPNs induces phase shift

**Figure 9.**
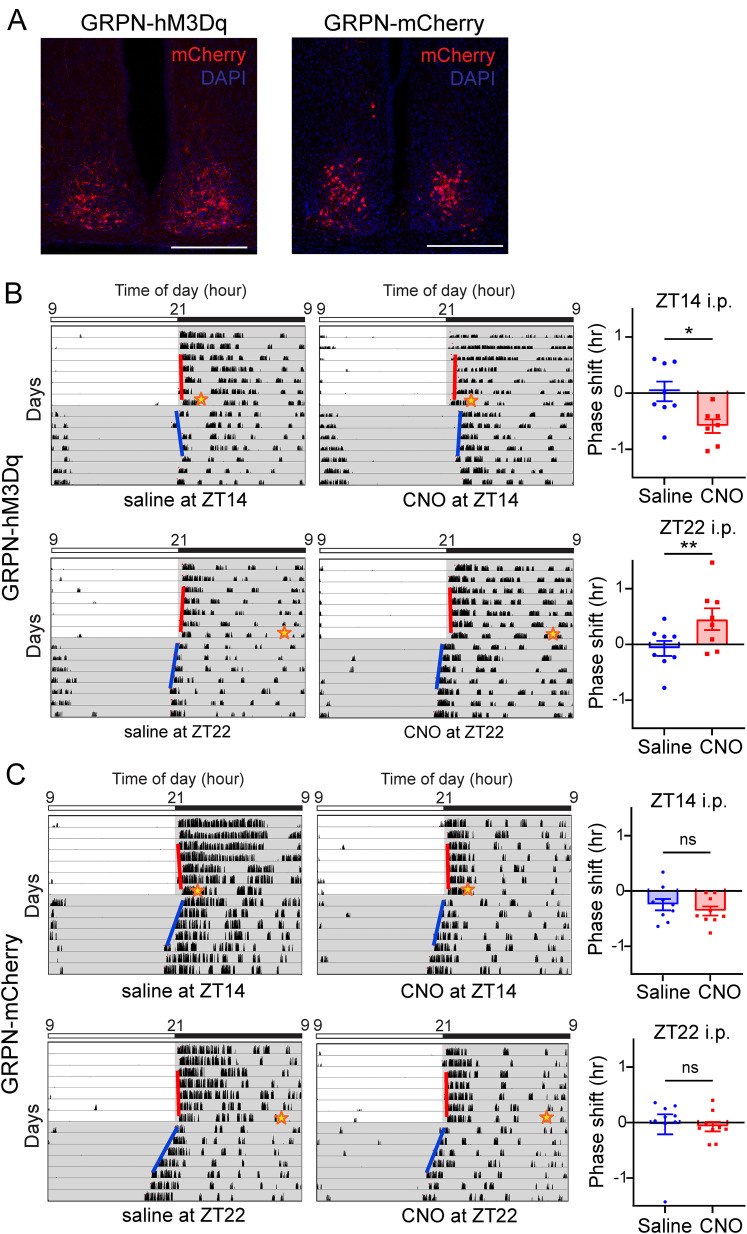
Stimulating the SCN GRPNs at different time points with DREADD-hM3Dq. ***A***, Representative images of SCN of each group showing the expression of mCherry (red) and DAPI. Scale bar is 250 μm. ***B***, Left side depicts the representative actograms of GRPN-hM3Dq mice before and after CNO or saline injected intraperitoneally. Bar on top displays time of day and lighting condition. Right side depicts graphs comparing the phase shift from saline or CNO injected intraperitoneally at either ZT14 or 22 (*n* = 7 for saline and CNO; *p* = 0.0343* for comparison of phase shift for i.p. at ZT = 14, *p* = 0.0030** for comparison of phase shift for i.p. at ZT = 22; paired Student's *t* test). ***C***, Left side depicts the representative actograms of GRPN-mCherry mice before and after CNO or saline injected intraperitoneally. Bar on top displays time of day and lighting condition. Right side depicts graphs comparing the phase shift from saline or CNO injected intraperitoneally at either ZT14 or 22 (*n* = 7 for both control and GRPNx; *p* = 0.9773 for ZT = 14 light pulse, *p* = 0.2935 for ZT = 22 light pulse; paired Student's *t* test). Red line in actogram indicates the onset fit determined by ClockLab before light pulse, and blue line indicates onset fit after light pulse.

Although GRPNx did not affect photic entrainment, it is possible that the lack of effect observed is due to compensation by other SCN neurons such as VIPNs. Since we found that SCN GRPNs immediately reacted to photic retinal stimulation (Fig. 8*B*), we examined whether the manipulation of these neurons induces a phase shift in the behavioral rhythm. After we expressed stimulatory DREADD hM3Dq in SCN GRPNs of *Grp-iCre* KI mice (GRPN-hM3Dq; Fig. 9*A*), CNO was injected at either ZT14 or 22 to examine phase shifts. We found that mice expressing hM3Dq in SCN GRPNs displayed a robust phase shift from CNO injection compared with saline injection (*n* = 7 for saline and CNO; *p* = 0.0343* for comparison of phase shift for i.p. injection at ZT = 14; *p* = 0.0030** for comparison of phase shift for i.p. injection at ZT = 22; paired Student's *t* test; Fig. 9*B*). Thus, we concluded that stimulating SCN GRPNs induced a phase shift.

As a control, mCherry was expressed in the SCN of *Grp-iCre* KI mice (GRPN-mCherry; Fig. 9*A*). The aforementioned experimental procedure was repeated for the GRPN-hM3Dq group. We found that there was no significant difference in phase shift between CNO and saline injections (*n* = 9 for saline and CNO; *p* = 0.3727 for comparison of phase shift for i.p. injection at ZT = 14, *p* = 0.8195 for comparison of phase shift for i.p. injection at ZT = 22; paired Student's *t* test; Fig. 9*C*). This proved that the phase shift observed in the above experiment was elicited by the activation of SCN GRPNs.

## Discussion

### Input and output organization of GRP-producing neurons in the SCN

In this study, we used *Grp-iCre* KI mice to elucidate the specific roles of GRP-producing neurons (GRPNs) in the SCN in the regulation of circadian rhythms and photic entrainment. Anterograde and retrograde tracing revealed that SCN GRPNs predominantly project to the thalamus and hypothalamus, with notable targets including the BNST, PVH, dSPZ, and DMH, while largely avoiding regions such as the VMH and Arc. The BNST contains GABAergic neurons that promote wakefulness (Kodani et al., 2017), suggesting that the GRPN projections to this region may influence sleep–wake regulation. The PVH contains corticotropin-releasing hormone (CRH)-producing neurons as part of the hypothalamic-pituitary-adrenal (HPA) axis (Spencer and Deak, 2017), and recent work has shown that SCN VIPNs projecting to these CRH neurons help regulate glucocorticoid rhythms (Jones et al., 2021). Since GRPNs constitute a subset of VIP neurons, it is plausible that GRPNs projecting to the PVH contribute similarly. Additionally, the dSPZ and DMH are key for body temperature rhythms (Lu et al., 2001), suggesting that GRPN projections may coordinate thermoregulatory signals. These anatomical findings hint at functions for GRPNs beyond photic entrainment.

Inputs to GRPNs were found to be primarily from within the SCN, supporting the concept of an intrinsic regulatory mechanism within the SCN that governs the functions of GRPNs. Because previous studies have reported that the retinal projections to the SCN terminate on GRPNs (Tanaka et al., 1997), we have also sampled the retina of some of the mice. However, we could not detect any GFP-positive neurons in the retina in our rabies virus-mediated tracing. In addition to the fact that the intrinsically photosensitive retinal ganglion cells (ipRGCs), which are known to project to the SCN, are scarce (a few percent of the retinal ganglion cells), we utilized rabies virus with a limited efficiency for retrograde transmission to reduce the nonspecific labeling. Thus, it is possible that we could not detect the direct input to the GRPNs from the retina simply due to experimental limitation. On the other hand, we demonstrated that GRPNs receive input from both core and shell neurons in the SCN. It is possible that ipRGCs in the retina project to other neurons in the core (such as VIPNs) and then relay the photic information to the GRPNs for light entrainment. The neurons in the shell that project to the GRPNs may play a different role. AVP-producing neurons in the shell serve as the pacemaker neurons for the SCN and relay rhythmic information to the core (Mieda et al., 2015; Shan et al., 2020). Thus, it is possible that the projections from the shell neurons to GRPNs are transmitting synchronizing signals to the GRPNs. These findings highlight the anatomical and functional specificity of SCN GRPNs in circadian network organization.

### Robust response and functional role of SCN GRPNs in photic stimulation and phase shifting

Our findings demonstrate that SCN GRPNs respond robustly to photic stimulation, as evidenced by increased intracellular calcium activity during retinal exposure to white light. While previous studies have reported an increase in c-Fos expression in GRPNs following retinal light exposure (Earnest et al., 1993), our study is the first to capture the acute (within seconds) response of GRPNs to retinal stimulation. Notably, the excitation of GRPNs using DREADD manipulation was sufficient to induce a phase shift in mouse behavioral rhythms. GRPN activation caused time-dependent phase delays in the early night and phase advance in the late night, strongly suggesting their involvement in the photic phase shift in vivo. Notably, despite being a small neuronal population within the SCN, GRPNs are capable of inducing robust phase shifts observable at the phenotypic level (Fig. 9).

However, despite these physiological responses and a potential phase-shifting function, our behavioral analyses indicated that neither GRP deficiency nor ablation of SCN GRPNs significantly affected circadian rhythm generation or photic entrainment. This suggests that other neuronal populations compensate for the GRPN dysfunction. The SCN contains other subtypes of neurons such as VIP-producing neurons (VIPNs), which are also implicated in photic entrainment (Jones et al., 2018). While GRPNs and VIPNs overlap, VIPNs are far more abundant, with ∼20–30% of VIPNs being GRP positive (Moore et al., 2002; Wen et al., 2020). Therefore, even in the absence of GRPNs, the remaining VIPNs play a compensatory role in conveying photic information to induce photic entrainment. Interestingly, the GCaMP signals in GRPNs and VIPNs displayed different kinetics from retinal light exposure, although we need to consider the differences in experimental conditions in our study and the VIPN study (Jones et al., 2018). VIPNs showed a long-lasting increase in Ca^2+^ signal even after the light exposure, while GRPNs’ response was acute and transient, suggesting their distinct roles. Thus, the quick light response of GRPNs may trigger the release of GRP and modulate the neuronal activity of VIPNs expressing GRP receptor. It is possible that this fine-tuning effect of GRPNs could not be detected in the condition of our behavioral experiments.

Although GRPNs do not appear essential for photic entrainment, their ability to independently induce phase shifts highlights a potential role as an auxiliary or modulatory node in the SCN network. This capacity may serve to enhance phase adjustment under specific conditions, such as rapid jet lag, seasonal light changes, or during aging, when SCN network synchrony weakens. Furthermore, the temporally precise and transient response of GRPNs suggests a potential role in signal gating or threshold modulation for downstream effectors, possibly interacting with VIP or AVP neurons in a context-dependent manner.

### Discrepancies in GRP/GRPR knockout phenotypes and potential compensatory mechanisms in SCN regulation

Compared with wild-type controls, GRP-deficient mice did not exhibit differences in locomotor activity rhythms or phase shifts in response to light pulses (Fig. 3). This finding contrasts with that of a previous study in which *Grp* receptor knock-out resulted in a slight attenuation of the phase shift and reduced induction of the *Per* gene in the dorsal SCN. This discrepancy may stem from differences in mouse genotypes (*Grp* vs *Grp* receptor KO) or variations in experimental conditions, such as the age of mice or the wheel-running setup. It is also possible that neuromedin B, another endogenous ligand that binds to GRPR, contributes to photic entrainment and plays a compensatory role in the absence of GRP. Our study is consistent with a previous study in which GRP deficiency did not significantly affect the locomotor activity rhythm. Thus, it was deduced that GRP is not involved in the generation of circadian rhythms in the SCN. But, considering the light response of GRPNs, its role in photic modulation of the circadian rhythms, such as light entrainment, light masking, or photoperiodism, rather than generation of circadian rhythms, was implied.

Our study highlights the nuanced roles of the SCN GRPNs in circadian regulation. Although they are not essential for the generation of circadian rhythms or photic entrainment, their activation can influence phase shifts. These findings provide new insights into the specific contributions of GRP-producing neurons within the SCN and open new avenues for investigating their roles in circadian biology. Further studies are warranted to explore the underlying mechanisms through which GRPN activation modulates phase shifts and delineate their potential interactions with other SCN neuronal populations involved in circadian regulation.

### Broader implications and future directions

Our results position GRPNs as key modulators, rather than essential pacemakers, within the SCN circuitry. Their projection targets—particularly those involved in arousal, hormonal rhythms, and thermoregulation—suggest roles beyond light-based entrainment, including potential involvement in behavioral state transitions and homeostatic adaptations. Notably, the BNST and PVH are also implicated in mood and stress-related disorders. Another possible role of the acute and transient light response in GRPNs is the regulation of contagious itch behavior rather than entrainment, as one study proposed that the SCN GRPNs convey the visual cues from the retina to induce itch behavior (Yu et al., 2017; Gao et al., 2022). Further studies should clarify the role GRPNs play in each of the phenomena.

While our study demonstrates that direct manipulation of SCN GRPNs can induce phase shifts, the specific neuropeptides mediating this effect remain to be identified. It would be interesting to zero in on the key neuropeptide in the GRPNs through chemogenetic/optogenetic manipulation of GRPNs in knock-out mice (e.g., VIP knock-out, GRP knock-out). Beyond circadian timing, the downstream targets of GRPNs—such as the BNST, dSVZ, and PVH—are also implicated in regulating arousal, body temperature, stress responses, and mood. These connections suggest that SCN GRPNs could serve as a conduit for translating environmental light cues into broader neuroendocrine and behavioral outputs. Understanding this pathway could illuminate the mechanisms by which circadian misalignment contributes to affective and metabolic disorders and highlight novel targets for therapeutic intervention in conditions such as depression, seasonal affective disorder, or shift work-related health issues.
